# A Foreign Body Lodged in the Glottis of a Toddler for a Prolonged Time: Anatomical Considerations and Review of the Literature

**DOI:** 10.7759/cureus.44489

**Published:** 2023-08-31

**Authors:** Anastasios K Goulioumis, Stamatios Peridis, Emmanouel Koudmnakis, Ioannis Athanasopoulos

**Affiliations:** 1 Otolaryngology - Head and Neck Surgery, Karamandaneio Pediatric Hospital, Patras, GRC; 2 Otorhinolaryngology, Private Practice, Rhodes, GRC; 3 Otorhinolaryngology, Mitera Hospital, Athens, GRC; 4 Otolaryngology - Head and Neck Surgery, Pediatric Center of "Iatriko Athinon" Hospital, Athens, GRC

**Keywords:** pediatric emergency, bronchoscopy, aspiration, laryngeal foreign body, foreign body

## Abstract

Our scope is to present the unusual case of an impacted foreign body in a child's larynx for a prolonged period due to recurrent misdiagnosis and review the literature emphasizing the laryngeal foreign body.

A toddler girl from a rural region was initially referred to a primary pediatric care center due to a sudden choking episode. The mother made an unsuccessful attempt to pull out a possible foreign body by blind finger sweeping. After 22 days of recurrent misdiagnosis and unsuccessful conservative therapies, the child developed hoarseness of voice and dyspnea during physical exertion. The patient underwent a flexible nasopharyngolaryngeal endoscopy, which observed a foreign body in the glottis, and an emergency microlaryngoscopy.

Persistence of laryngeal symptoms in a child with a sudden choking episode should always raise the suspicion of a respiratory tract foreign body impaction. The most appropriate therapeutical approach is rigid bronchoscopy under general anesthesia.

## Introduction

A possible foreign body (FB) aspiration is a typical case presentation to the emergency department of pediatric hospitals. An actual aspiration occurs in 3/10,000 children per year [1]. The mortality of this condition reaches 2.5% of the cases, making it the third leading cause of death in children from one to four years old [2].

Children, especially before the age of three, tend to investigate their environment by putting objects in their mouths, which is considered a normal developmental stage [1]. Additionally, it is not unusual for them to play and run while chewing. The previous is more common among male toddlers rendering them more vulnerable to aspiration than females in a 2:1 ratio [3]. Considering the relatively narrow laryngeal inlet, the underdeveloped posterior dentition, and the less mature swallowing and coughing reflex compared to adults, aspiration and sequela are possible concerns after any choking episode in children [1].

This current report presents the unusual case of a hairpin lodged in the larynx for a prolonged period due to recurrent misdiagnosis. Additionally, this report reviews the literature, including the main issues concerning FB aspiration, emphasizing the FB in the larynx and the anatomical considerations determining the obstruction level and the symptomatology.

## Case presentation

After the approval of our tertiary pediatric hospital's Institutional Review Board (IRB), we present the following clinical case of an FB aspiration in a toddler girl. The patient living in a rural region of Greece was initially referred to a primary pediatric care center due to a choking episode. The parents heard their child suddenly coughing, and the mother made an unsuccessful attempt to pull out a possible FB by blind finger sweeping. The girl remained asymptomatic after the initial coughing and a subsequent vomiting episode. The physical examination was normal, without any signs of upper or lower respiratory tract infection. The chest and abdominal X-rays performed were also unremarkable, but mistakenly they did not contain the larynx.

Over the following days, the girl gradually presented hoarseness of voice without being accompanied by dyspnea, coughing, or stridor. The pediatricians attributed the condition to a laryngeal injury caused by the mother's attempts to pull out a possible FB and the girl was treated with oral corticosteroids and antibiotics for 10 days.

**Figure 1:**
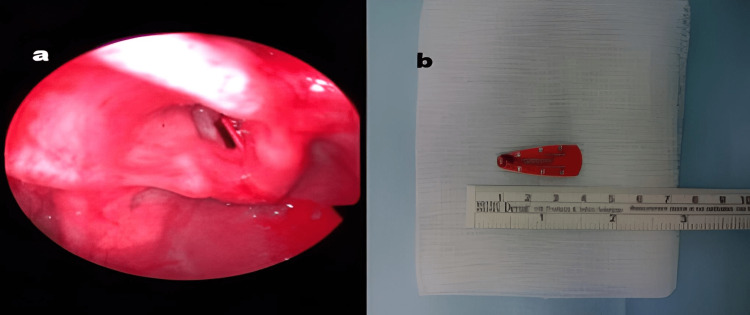
(a) A metallic hairpin stacked in the sagittal plane in between false vocal cords at the anterior commissure. (b) The metallic pin measured 3 cm.

As the hoarseness of her voice did not improve, she was referred to a private otorhinolaryngologist. A flexible nasopharyngolaryngeal endoscopy was undertaken, and the girl was diagnosed with glottic inflammation. The patient had been prescribed another course of antibiotics and inhaled corticosteroids for one more week. Although an initial improvement was noticed, the girl developed deteriorated hoarseness of voice and dyspnea during physical exertion on day 22. The child was referred to the otorhinolaryngology department of Agia Sofia Pediatric Hospital of Athens. On the same day of admission, the patient underwent a flexible nasopharyngolaryngeal endoscopy, where an FB was observed in the glottis. After that, an emergency micro-laryngoscopy and bronchoscopy with a rigid bronchoscope under general anesthesia were undertaken. A 3 cm metallic hairpin was removed in the sagittal plane between false vocal cords and subglottis (Figure 1).

The girl was discharged on the second postoperative day free of symptoms with inhaled corticosteroids for seven days and simple analgesia, as required. A postoperative follow-up with a flexible nasopharyngolaryngeal endoscopy was performed after two weeks, with no pathological signs observed and other symptoms reported.

## Discussion

The clinical presentation of an FB aspiration case depends on several parameters, primarily on the object's size, which determines the level of the airway where it may get stuck. Additionally, clinical appearance depends on the object's shape, which can induce a valve mechanism as air passes during inspiration when airways also dilate but cannot be exhaled [4]. Other parameters crucial for the clinical appearance are the irritating or inert nature of the object, the patient's age that determines the diameter of the airways, the possible preceding therapeutic interventions to which the airway may initially respond, and the time interval between the choking episode and the examination [1].

The clinical findings in the case of aspiration are progressing in three stages. Initially, the child appears with paroxysmal coughing, which lasts for several minutes. The choking episode is witnessed by a parent in only two-thirds of the cases [4]. Subsequently, a remission of the acute signs occurs before complications appear in the third stage. The remission-of-symptoms stage may last for hours to weeks, depending on the nature of the FB. An organic FB that may swell and induce mucosa inflammation is anticipated to have a more sudden progression to complications than an inert FB, like plastic [3]. In the long term, expected complications of an FB in the bronchus include atelectasis, pneumonia, abscess, granuloma formation, bronchiectasis, hemoptysis, and pneumothorax [5].

In almost 80% of the cases of aspirations, a food article, usually nuts, is implicated [1]. Notably, 85% of the aspired FBs are stacked in the main bronchi [3]. Although in adults, it is more usual to locate the FB in the right main bronchus primarily due to the geometry of the carina and the greater inclination of the left bronchus due to the position of the heart, such a case does not always apply to children [1]. The main bronchi are the most typical anatomical sites of the airway where an aspired FB is lodged; however, depending on the size and shape of the object, it may get stuck at any level of the airway, manifesting different signs and symptoms.

FBs too large to pass through the laryngeal inlet or with irregular edges tend to get lodged in the larynx. Laryngeal FBs are only 1-7% of the cases of aspiration, primarily in children younger than one year of age [6]. To become lodged in the larynx, an object must have at least one diameter equal to or slightly larger than the glottis or subglottis. The tendency for an FB to get stuck high in the airway also depends on the phase of the respiratory circle when the aspiration occurs. Thus, at the end of inspiration, the airway and atmospheric pressure are near equilibrium, and there is less suction effect to impale the object [6]. Thin and flat triangular objects like metallic safety pins can also get stuck in the sagittal plane between vocal cords or in the narrow, conical-shaped subglottic region. Triangular aerodynamic objects or objects with a lumen cause only moderate respiratory distress because air exchange around or through them is almost normal. Although the victim of aspiration coughs immediately, the cough is ineffective in expelling the FB due to the object's relative lack of surface upon which the air pressure can exert force. Subsequent mucosa edema leads to an even more firm object's impaction between the anterior and posterior commissures [6].

Diagnosis of FB aspiration may be based on three pillars: the history of an evidenced choking episode, the clinical examination with chest auscultation, and the radiology exams [3]. Many centers combine the results of these findings in diagnostic algorithms, which, however, have a diagnostic accuracy that is not surpassing 68% [5]. Evaluation of the patient becomes even more complicated when other conditions co-exist, like airway viral infection or asthma. At the same time, FB aspiration should always be included in the differential diagnosis when recurrent tracheobronchitis, asthma, pneumonia, and lung abscess episodes occur [3].

The choking episode is witnessed in only half of the cases [5]. On the other hand, even if the evidence of a choking episode is enough for a clinician to include aspiration in the differential diagnosis, more than half of children with evidenced choking episodes did not have an FB in their airways after a bronchoscopy [5]. Thus, a witnessed event cannot be considered a good predictor of the presence or absence of any airway FB.

In the chest auscultation, there are no pathognomonic signs; however, the combination of persistent coughing despite medical therapy, unilateral reduction of breathing sounds, localized expiratory stridor, and timely alteration in the auscultation findings had a positive predictive value for FB aspiration of 90% [7]. On the other hand, even in confirmed cases of aspiration, as mentioned earlier, the combination of the classical signs manifests typically in at most 35% of the cases [7]. Notably, 6% of the cases of aspiration at the initial physical examination may appear asymptomatic, rendering reexamination crucial in due course when history indicates aspiration [5].

The chest X-ray may depict an FB only in 4-16% of the cases when it is radiopaque [8]. In the possible case of a radiolucent FB, an early chest X-ray has a sensitivity of 70% and a specificity of 45% [5]. Nevertheless, the initial X-ray is valuable as a reference to compare with subsequent X-rays. Imaging exams should include X-rays of the chest and neck. The detailed examination of a neck X-ray is critical to exclude an FB lodged in the larynx [9]. X-rays should be conducted in the inspiratory and expiratory phases to enhance the most typical finding of the initial stage, which is the appearance of emphysematous areas due to the valve phenomenon induced by the FB [5]. X-rays performed in a later stage are more likely to exhibit atelectatic areas or consolidations [10]. Probably, it is more difficult to attain an inspiratory/expiratory X-ray in children younger than five years old but particular positioning of the child, like decubitus position, may lead to corresponding findings. However, some studies support that adding decubitus to standard views does not show clinical benefit [7]. Fluoroscopy, 3D virtual bronchoscopy, and chest CT are additional options for FB aspiration diagnosis [10]. More specifically, CT seems to be a more sensitive modality than conventional radiography, especially in the case of a residual FB after bronchoscopy, which occurs in 1-8% of the cases [11]. Many centers have embedded CT in their diagnostic algorithms to reduce negative bronchoscopies. An unnecessary bronchoscopy under general anesthesia exposes patients to potential complications. Moreover, the need for general anesthesia, which has a not yet clear neurocognitive impact on children younger than three years old, would discourage unnecessary bronchoscopies [7]. CT is a quick procedure that may not need sedation and can be arranged in a few hours, showing applicability for triage in community hospitals where otolaryngologists are unavailable [11]. However, radiation issues, as well as false positive findings due to mucous plugs and false negative findings in the case of thin and small FBs less than 3 mm, render CT’s selection as the first choice exam somewhat questionable [10].

Notably, flexible fiberoptic endoscopy is the only means that can directly confirm a possible FB in the airways. Many centers utilize this method as a diagnostic tool just before proceeding to rigid bronchoscopy [12]. Laryngeal endoscopy is also an essential diagnostic tool for a suspected laryngeal FB. Given the compromised and unstable airway in such a case, laryngeal endoscopy should always be conducted under the proper supervision of the patient and in the presence of a doctor capable of performing an emergency tracheostomy in a child if needed.

Focusing on laryngeal FB, the spectrum of presentations varies widely, ranging from sudden death to hoarseness of voice and accidental findings during a routine investigation [13]. The most common presentation of a laryngeal FB is sudden hoarseness, croupy cough, and biphasic stridor [13]. However, the mucosa of the larynx adapts to the presence of the FB, and the imminent irritating symptoms may have subsided by the time of admission, especially if the acute episode was not witnessed. Only a minority of patients present with symptoms like aphonia, odynophagia, hemoptysis, wheezing, cyanosis, and varying degrees of dyspnea [13]. Sometimes, a laryngeal FB may mimic viral laryngitis or epiglottitis. The symptoms may even improve with antibiotics, bronchodilators, and steroids, like in our case, leading to prolonged misdiagnosis, even for weeks [14]. A multivariate analysis of laryngeal FB cases [13] showed that the interval between aspiration and admission for more than five days, unwitnessed aspiration episode, absence of biphasic stridor or aphonia, and negative X-ray manifestations were retained as independent risk factors for misdiagnosis. Surprisingly, two seemingly obvious risk factors, radiolucent and small-size FBs, did not show any significant statistical correlation with misdiagnosis.

Parents usually instinctively make unsuccessful attempts to remove the FB by blind finger sweeping, which probably impacts a loose FB tightly into the larynx or induces trauma and bleeding, thus transforming partial into a complete obstruction [14]. Blind finger maneuver in children will probably complicate the situation, while it could rarely be beneficial in adults. Thus, it has been vastly replaced by the Heimlich maneuver [15].

The most appropriate therapeutical approach for FB aspiration is rigid bronchoscopy under general anesthesia [3]. The decision for a bronchoscopy should be based on the history of a choking episode, the auscultation findings, and secondarily on the X-ray results. Clinicians should always have a high index of clinical suspicion for aspiration and a low decision threshold to perform a bronchoscopy. Consequently, approximately one-third of bronchoscopies are expected not to discover an FB [16]. On the other hand, 14% of the bronchoscopies for an FB are delayed for a week or more [17]. It is essential to have a variety of appropriate equipment consistent with the child's age [18]. Bronchoscopy is an acute procedure but is usually not emergent. It should only be attempted when an experienced team of otolaryngologists, anesthesiologists, and nurses is present [18] since it is a procedure with mortality that reaches 0.4% and escalates in delayed cases [5]. Even for two days, a delayed presentation has also been related to more copious bronchoscopies vulnerable to complications and longer hospital stays [17]. In 3% of bronchoscopies, patients may suffer from severe complications like hypoxia, arrhythmia with cardiac arrest, laryngeal trauma, bleeding, bronchial perforation, pneumothorax, pneumonic edema, and 15% of minor complications, which include teeth fracture and bronchospasm [12]. Ventilation is mediated through either the rigid bronchoscope or jet ventilation [18]. Spontaneous breathing and apneic techniques are additional options [18]. Close cooperation between the otorhinolaryngologist and anesthesiologist is essential to maintain the airway at all times. For the FBs in the distal bronchi or the bronchus of the upper lobes, a fiberoptic flexible bronchoscope with a working channel may be helpful [18]. Notably, many centers present good results with flexible fiberoptic bronchoscopy as a first-line therapeutic approach [12]. Currently, the standard flexible bronchoscopes have an outer diameter of 4 mm with a 2 mm working channel. They can be inserted through a laryngeal mask airway (LMA) or a tracheal tube [12]. Even thinner flexible bronchoscopes with a working channel have been developed as prototypes, though, at the expense of the variety of useful instruments that can be inserted through the working channel [19]. In 17% of the cases of aspiration, a repeated procedure may be attempted when the first bronchoscopy was unsuccessful [3]. Nevertheless, bronchoscopy may be inadequate in 2% of cases, and thoracotomy for bronchotomy, segmentectomy, or pneumonectomy may become necessary subsequent interventions [20].

## Conclusions

FB aspiration is a typical medical emergency an otorhinolaryngologist may encounter. An acute index of suspicion is always needed in the case of pediatric patients. Thus, focusing on laryngeal FB when there is a sudden onset of respiratory symptoms like hoarseness or stridor developing without an apparent reason in a child without a previous history of asthma, aspiration should be suspected. Radiologic and endoscopic examinations should then be conducted. However, prevention via education regarding nuts consumption and playing with small toys with parts that can be aspirated into the airway of a small child is the utmost way of avoiding aspirations, together with a public education program for applying basic life support principles to a victim of aspiration.
